# SPINK1 Overexpression Correlates with Hepatocellular Carcinoma Treatment Resistance Revealed by Single Cell RNA-Sequencing and Spatial Transcriptomics

**DOI:** 10.3390/biom14030265

**Published:** 2024-02-22

**Authors:** Chunyuan Yang, Limei Guo, Juan Du, Qiulu Zhang, Lingfu Zhang

**Affiliations:** 1 Institute of Systems Biomedicine, Department of Pathology, School of Basic Medical Sciences, Peking University Third Hospital, Peking University Health Science Center, Beijing 100191, China; yangchunhyuan@bjmu.edu.cn (C.Y.); dujuan122@bjmu.edu.cn (J.D.); zhangqiulu@bjmu.edu.cn (Q.Z.); 2 Department of General Surgery, Peking University Third Hospital, Beijing 100191, China; zhanglingfu@bjmu.edu.cn

**Keywords:** SPINK1, hepatocellular carcinoma, single cell RNA sequencing, spatial transcriptomics, CES2, CYP3A5, chemotherapy, targeted therapy, immune checkpoint inhibitor

## Abstract

Low efficacy of treatments and chemoresistance are challenges in addressing refractory hepatocellular carcinoma (HCC). SPINK1, an oncogenic protein, is frequently overexpressed in many HCC cases. However, the impact of SPINK1 on HCC treatment resistance remains poorly understood. Here, we elucidate the functions of SPINK1 on HCC therapy resistance. Analysis of SPINK1 protein level reveals a correlation between elevated SPINK1 expression and unfavorable prognosis. Furthermore, intercellular variations in SPINK1 expression levels are observed. Subsequent examination of single cell RNA-sequencing data from two HCC cohorts further suggest that *SPINK1*-high cells exhibit heightened activity in drug metabolic pathways compared to *SPINK1*-low HCC cells. High *SPINK1* expression is associated with reduced sensitivities to both chemotherapy drugs and targeted therapies. Moreover, spatial transcriptomics data indicate that elevated *SPINK1* expression correlates with non-responsive phenotype during treatment with targeted therapy and immune checkpoint inhibitors. This is attributed to increased levels of drug metabolic regulators, especially *CES2* and *CYP3A5*, in *SPINK1*-high cells. Experimental evidence further demonstrates that *SPINK1* overexpression induces the expression of *CES2* and *CYP3A5*, consequently promoting chemoresistance to sorafenib and oxaliplatin. In summary, our study unveils the predictive role of SPINK1 on HCC treatment resistance, identifying it as a potential therapeutic target for refractory HCC.

## 1. Introduction

Liver cancer ranks as the third cause of mortality of all malignancies, and 90% of liver cancers are hepatocellular carcinoma (HCC) [1,2]. The overall prognosis of HCC is heavily reliant on the tumor stage. Following the Barcelona Clinic Liver Cancer (BCLC) staging system, HCC patients are categorized into very early stage, early stage, intermediate stage, late stage, and terminal stage. Patients in the very early stage benefit from resection and local ablation, while those in the intermediate stage are suitable candidates for trans-arterial chemoembolization. Patients in the advanced stage are considered for systemic therapies [3,4]. Despite advances in targeted therapies and immune checkpoint blockade, the major obstacle to HCC treatment remains the low response rate [5]. Therefore, it is imperative to explore the mechanisms of chemoresistance to enhance treatment effectiveness and extend the prognosis for HCC patients.

Serine peptidase inhibitor Kazal type 1 (SPINK1) is a secretory protein that plays important roles in various physiological processes, including pancreatic functions, sperm maturation, and skin barrier homeostasis [6]. Notably, SPINK1 also assumes critical functions in cancer pathogenesis, and has been identified as dysregulated in many tumor types, including HCC [7]. In the context of HCC, the expression level of SPINK1 has been observed to escalate with the progression of HCC [8]. Elevated levels of serum SPINK1 show promise as a diagnostic indicator for HCC [9].

Mechanistically, SPINK1 has been implicated in tumor cell proliferation and metastasis through the MEK/ERK signaling pathway [10,11]. Moreover, a high expression of SPINK1 is associated with the presence of portal vein tumor thrombus and is indicative of a poorer prognosis for patients [12,13]. However, the precise impact of SPINK1 on HCC therapy remains unclear.

Here, employing a combination of bioinformatics and experimental validations, we elucidate the pivotal roles of SPINK1 in conferring therapy resistance in HCC. We initially assembled an HCC cohort and performed immunohistochemistry (IHC) to detect the histological heterogeneity of SPINK1 expression. The application of single cell RNA-sequencing (scRNA-seq) not only mitigated the confounding effects of tumor heterogeneities, but also facilitated the delineation of SPINK1 functions at the single cell level. This approach enabled the identification of active drug detoxification pathways in cells with elevated SPINK1 expression.

Furthermore, the incorporation of spatial transcriptomics (ST) allowed for in situ validations for SPINK1 functional mechanisms by revealing the co-localization of SPINK1 with key regulators of drug metabolism. Collectively, our findings shed light on the crucial regulatory functions of SPINK1 in fostering treatment resistance in HCC. These insights not only enhance our understanding of the underlying mechanisms but also present SPINK1 as a promising therapeutic target for overcoming HCC treatment challenges.

## 2. Materials and Methods

### 2.1. SPINK1 Protein Expression Level Detection in HCC

The expression data of SPINK1 were obtained from the Clinical Proteomic Tumor Analysis Consortium (CPTAC) database “https://proteomic.datacommons.cancer.gov/pdc/browse (accessed on 8 February 2024)” [14]. The original liver cancer data, including experimental protocols and analysis methods, were sourced from a previously published HCC cohort [15] (cohort 1, Supplementary Table S1).

### 2.2. HCC Cohort Collection

Our study was approved by the Ethics Committee of Peking University Third Hospital. All research was conducted in accordance with both the Declarations of Helsinki and Istanbul. Liver cancer patients who underwent surgery at the Department of General Surgery of the Peking University Third Hospital were enrolled based on the following criteria: (1) pathologically confirmed HCC; (2) no history of other malignancies; and (3) no anti-cancer therapy before surgery. All patients were provided with comprehensive information and their identities were kept confidential.

A total of 58 surgical specimens from primary HCC cases, resected at the Peking University Third Hospital, constituted cohort 2. For each tumor, the gross and microscopic findings were meticulously reviewed by two pathologists in a blinded fashion. Tumors were categorized based on histological type, differentiation grade, and stage following the WHO Classification of Tumors (2019).

### 2.3. IHC Staining

Formalin-fixed and paraffin-embedded 4-µm tissue sections were used for IHC staining. In a stepwise process, sections underwent dehydration with graded concentrations of ethanol and were immersed in 3% hydrogen peroxide for 15 min to block endogenous peroxidase activity. Antigen retrieval was performed by heating the sections for 2 min in a pressure cooker using 0.01 M citrate buffer (pH 6.0). Subsequently, the sections were incubated overnight at 4 °C with primary antibodies against SPINK1 (Abcam, Cambridge, UK). For the secondary antibody, either the histidine Envision Chem Detection Kit (Agilent Dako, Santa Clara, CA, USA) or GTVision^TM^ Double Staining Detection System (Agilent Dako, Santa Clara, CA, USA) was used. The chromogen used was 3,3-diaminobenzidine/hydrogen peroxide. As a negative control, PBS was substituted for the primary antibody.

### 2.4. Analysis of scRNA-Seq Data

Our scRNA-seq data from HCC samples were sourced from the Gene Expression Omnibus (GEO) database (GSE156337 and GSE149614). We used a two-step method to analyze the functions of SPINK1 in tumor cells of HCC.

In the first step, analysis of cells in these two cohorts involved normalization, reduction, unsupervised clustering, and annotation. LogNormalize was used for normalization, and PCA served as the reduction method. Standard procedures as described in https://satijalab.org/seurat/ (accessed on 8 February 2024) were followed for cell clustering. Cell types were annotated using SingleR (V1.4.1) with the reference “Human Primary Cell Atlas Data Labels”. The resulting cell identities were classified as B cell, endothelial cell, hepatocyte, fibroblast, myeloid cell, and T/NK cell.

In the second step, the hepatocyte cluster was isolated, and cells from normal tissues were filtered out. Only cells from tumor tissues were retained, assuming these malignant hepatocytes represented tumor cells. Subsequently, using unsupervised clustering, these tumor cells were re-clustered into 39 sub-clusters in cohort 3 and 38 sub-clusters in cohort 4. In cohort 3, tumor sub-clusters numbered 5, 7, 8, 11, 12, 14, 17, 18, 19, 20, 21, 22, 23, 24, 27, 28, 29, 30, 31, 32, and 33 exhibited elevated *SPINK1* expression, leading us to integrate these sub-clusters as *SPINK1*-high HCC cells. The remaining sub-clusters were consolidated as *SPINK1*-low HCC cells. In cohort 4, tumor sub-clusters numbered 2, 5, 6, 7, 8, 12, 14, 15, 17, 18, 19, 21, 22, 24, 27, 30, 32, 34, and 35 demonstrated high *SPINK1* expression, promoting the integration of these sub-clusters as *SPINK1*-high HCC cells. The remaining sub-clusters were integrated as *SPINK1*-low HCC cells.

### 2.5. Gene Set Enrichment Analysis and Gene Set Variation Analysis

We compiled multiple drug resistance pathways, such as the cisplatin resistance pathway, fluorouracil resistance pathway, gefitinib resistance pathway, doxorubicin resistance pathway, gemcitabine resistance pathway, docetaxel resistance pathway, trabectedin resistance pathway, and tamoxifen resistance pathway, from MSigDB (http://www.gsea-msigdb.org/gsea/msigdb (accessed on 8 February 2024)). To assess the enrichment of DEGs for *SPINK1*-high cells within these gene sets, we employed Gene Set Enrichment Analysis (GSEA).

Furthermore, Gene Set Variation Analysis (GSVA) was utilized to assess distinct metabolic states in *SPINK1*-high and *SPINK1*-low cells through the R package GSVA. Significantly perturbed pathways were identified through the R package Limma, with a Benjamini–Hochberg-corrected *p*-value threshold set at ≤0.01.

### 2.6. Drug Sensitivity Analysis

The gene expression level of *SPINK1* in various cell lines and their respective drug sensitivities were obtained from CellMiner (NCI-60) (https://discover.nci.nih.gov/cellminer (accessed on 8 February 2024)). The correlation between *SPINK1* expression levels and drug sensitivities were assessed using R package ggplot2. The statistical significance of these correlation was determined through Pearson’s correlation analysis, with a significance threshold set at *p* ≤ 0.05.

### 2.7. Analysis of ST Data

ST data were utilized to identify the co-expression pattern of *SPINK1* with *CES2* and *CYP3A5*. The significant of gene co-expression was determined through Spearman correlation analysis, and *p* values ≤ 0.05 were considered to be significantly co-expressed.

### 2.8. Quantitative Real-Time PCR

After isolation using TRIzol reagent (Invitrogen, Cat# 15596026), mRNA was reverse transcribed into cDNA using all-in-one 5 MasterMix (abm, Cat# G592), followed by quantitative real-time PCR analysis (TransGene, Cat# AQ132-21). Quantitative real-time PCR primers include *CES2* (F, CATGGCTTCCTTGTATGATGGT; R, CTCCAAAGTGGGCGATATTCTG), *CYP3A5* (F, GGTGGTGATTCCAACTTATGCT; R, GCGTGTCTAATTTCAAGGGGA), and *ACTB* (F, TGACCCAGATCATGTTTGAG; R, TTAATGTCACGCACGATTTCC). The Ct value of each detected cDNA was normalized to that of ACTB.

### 2.9. SPINK1 Overexpression and Western Blot Analysis

To stability overexpress *SPINK1* in PLC/PRF/5 cells, pCDH CMV puro (control) or pCDH SPINK1 (overexpression) plasmids, coupled with pMD.2G and pAX.2 plasmids, were co-transfected into HEK293 cells. 48 h later, the supernatant combined with polybrene (10 μg/mL) was transferred to the PLC/PRF/5 cell culture system and, 24 h later, the successfully transfected cells were screened using puromycin (5 μg/mL).

In Western blot analysis, PLC/PRF/5 cells were lysed in RIPA lysis buffer (50 mM Tris-HCl (pH = 7.4), 150 mM NaCl, 1 mM EDTA, 5 mM EGTA, 1% NP40, and 0.1% sodium deoxycholate) supplemented with a ‘cocktail’ of protease inhibitors. Cell lysates were subjected to SDS–PAGE and immunoblot analysis was performed using antibodies against GAPDH (Ray Antibody, Cat# RM2002) and SPINK1 (Abcam, Cat# ab206294).

### 2.10. Stable Short Hairpin RNA Knockdown of SPINK1

The shSPINK1 sequence was 5′-CCCTGTTGAGTCTATCTGGTA-3′. To generate shSPINK1 lentivirus, pMD.2G, pAX.2, and pLKO.1 were co-transfected into the HEK293 cells. Then, 48 h later, the supernatant combined with polybrene (10 μg/mL) was transferred to the *SPINK1* overexpressed PLC/PRF/5 cell culture system. After 24 h, the supernatant combined with polybrene (10 μg/mL) was once again transferred to the *SPINK1* overexpressed PLC/PRF/5 cell culture system to enhance the knockdown effectiveness.

### 2.11. Chemoresistance Analysis

PLC/PRF/5 cells, including control cells and *SPINK1* overexpression cells, were seeded into 96-well plates. Subsequently, these cells were exposed to sorafenib (at concentrations of 0, 1, 2, 4, 6, 8, 10, and 20 μM) or oxaliplatin (at concentrations of 0, 1, 2, 4, 6, and 8 μM) for 24 h, independently. Drug sensitivity was analyzed by measuring the survival cell proportions under each condition using CCK-8 assay (BeijingWJLZ biotechnology, Cat# WJ30025) in accordance with the manufacturer’s instructions. Survival cell proportions in both control group and *SPINK1* overexpression group are normalized to that in cells treated with 0 μM sorafenib or oxaliplatin, respectively.

To detect the effectiveness of SPINK1 knockdown on chemoresistance, control PLC/PRF/5 cells (con), *SPINK1* overexpressed PLC/PRF/5 cells (SPINK1-OE), and *SPINK1* overexpression followed by gene expression knockdown PLC/PRF/5 cells (OE + shSPINK1) were exposed to 8 μM sorafenib or oxaliplatin for 24 h. Survival cell proportion assessments were conducted as previously described.

### 2.12. Statistics

The comparisons of SPINK1 protein expression levels between tumor and non-tumoral normal tissue as well as the CCK-8 assay results were performed using unpaired Student’s *t*-test, and data are represented as means ± standard error of mean. Patient survival analysis was performed using the log-rank (Mantel-Cox) test. GSVA was conducted through a Kolmogorov–Smirnov-like random walk test. The GSEA was performed using the Wilcoxon–Mann–Whitney test. Drug sensitivity was analyzed using the Chi-square test. Gene co-expression of *SPINK1* with *CES2* and *CYP3A5* was performed using Spearman’s correlation analysis. All statistical analyses were performed by R 4.3.2 or GraphPad Prism 10, and differences were considered significant when *p* < 0.05.

## 3. Results

### 3.1. The Protein Level of SPINK1 Correlates with HCC Malignancy

**Figure 1:**
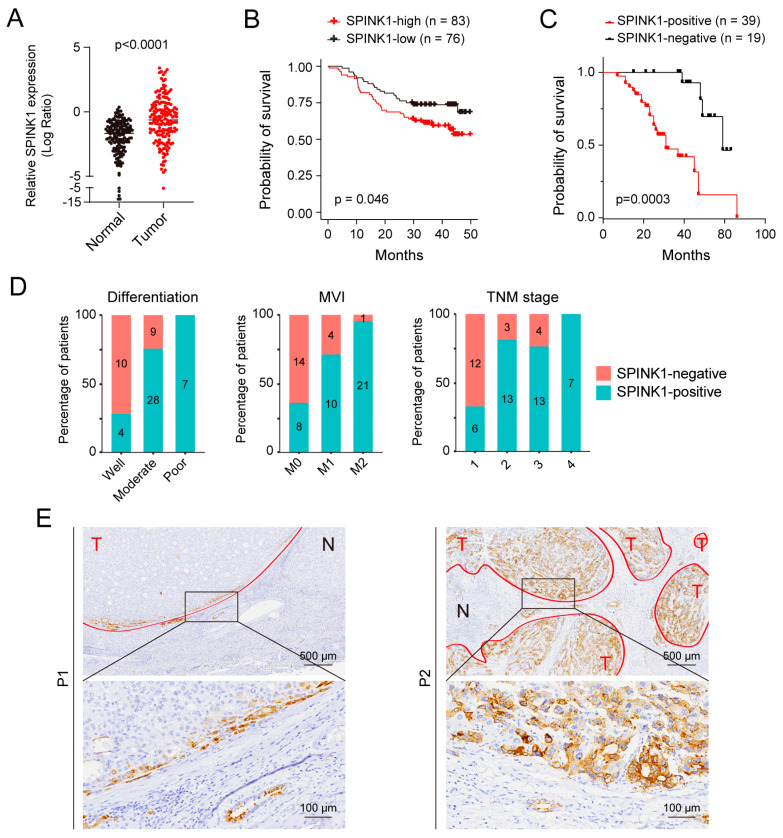
High level of SPINK1 expression indicates higher malignancy in HCC patients. (**A**) Dot plot illustrating the relative SPINK1 protein expression levels in HCC tumor (red) compared to normal tissues (black) in cohort 1. (**B**) Survival curve based on cohort 1 demonstrating the relationship between patient survival percentage and the expression levels of SPINK1. HCC patients with higher expression levels of SPINK1 showed shorter survival periods. (**C**) Survival curve based on cohort 2 demonstrating the relationship between patient survival percentage and SPINK1 expression levels. HCC patients with higher expression levels of SPINK1 showed shorter survival periods. (**D**) Stacked histograms depicting the differentiation state (left), microvascular invasion (MVI) state (middle), and TNM state (right) of patients with (blue) or without (red) SPINK1 expression in cohort 2. Number of patients in each group are labeled in graphs. (**E**) Representative IHC staining images of SPINK1 in two HCC samples. T, tumor; N, normal.

While numerous reports have highlighted the association between elevated *SPINK1* mRNA levels and worse HCC prognosis [16], investigations on SPINK1 protein levels are relatively scarce. To detect the protein expression level of SPINK1 in HCC patients, we searched SPINK1 protein expression levels in HCC cohort 1 (Supplementary Table S1) and found higher SPINK1 protein levels in tumor samples than peritumoral normal tissues (Figure 1A). Higher SPINK1 levels in HCC patients correlated with worse prognosis (Figure 1B), suggesting the critical functions of SPINK1 on HCC progression. To delve into the clinic-pathological relevance of SPINK1, we collected an HCC cohort consisting of 58 treatment-naïve patients (cohort 2, Supplementary Table S1) and performed IHC staining of SPINK1 on these samples. We found that patients with high protein levels of SPINK1 showed worse prognosis than those with low protein levels of SPINK1 (Figure 1C).

Through an assessment of the correlation between SPINK1 expression status and various pathological features, including tumor size, differentiation status, microvascular invasion (MVI), capsular invasion, virus infection background, TNM stage, and TP53 status (Table 1), we found that a higher SPINK1 protein level correlates with a poorer tumor differentiation status, MVI status, and higher TNM stage (Figure 1D). Intriguingly, the expression of SPINK1 in tumor nodules is not homogeneous (Figure 1E), suggesting cellular heterogeneity of SPINK1 expression levels.

### 3.2. Transcriptomic Profiles of SPINK1-High Tumor Cells Revealed by scRNA-Seq Data

**Figure 2:**
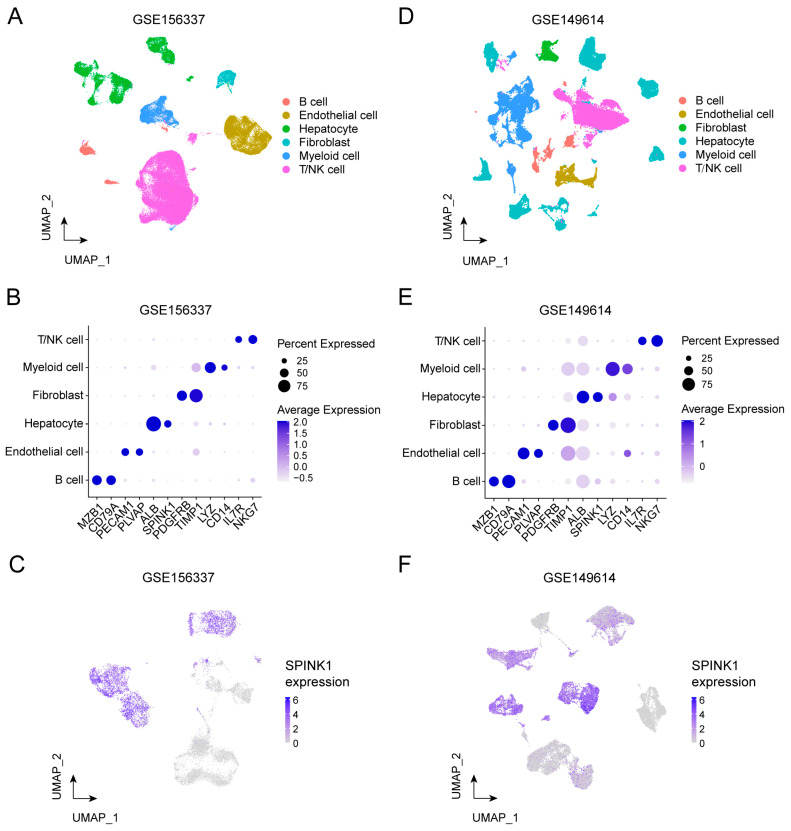
Transcriptomic profiles of *SPINK1*-high cells revealed by scRNA-seq data. (**A**) Uniform manifold approximation and projection (UMAP) plot of cohort 3 (GSE156337) divided by cell types. (**B**) Dot plot illustrating the marker gene expression levels of cell types in cohort 3 (GSE156337). (**C**) UMAP plot displaying the relative expression levels of *SPINK1* in cohort 3 (GSE156337). *SPINK1* expression level was colored from gray (low expression) to blue (high expression). (**D**) UMAP plot of cohort 4 (GSE149614) divided by cell types. (**E**) Dot plot illustrating the marker gene expression levels of cell types in cohort 4 (GSE149614). (**F**) UMAP plot displaying the relative expression levels of *SPINK1* in cohort 4 (GSE149614). *SPINK1* expression level was colored from gray (low expression) to blue (high expression).

To assess cellular transcriptomic heterogeneity and explore SPINK1 functions at the single cell level, we utilized two HCC scRNA-seq datasets, namely, cohort 3 and cohort 4 (Supplementary Table S1) [17,18]. Analyzing all cells in cohort 3 identified six cell clusters: B cell, endothelial cell, hepatocyte, fibroblast, myeloid cell, and T/NK cell (Figure 2A). Specifically expressed marker genes of each cell clusters further verified the accuracy of our cell type annotation (Figure 2B). Upon re-analysis of the tumor cell cluster in cohort 3, 39 sub-clusters were identified (Supplementary Figure S1A,B), among which *SPINK1* showed variable expression levels (Figure 2C). Given the elevated expression levels of *SPINK1* in sub-clusters numbered 5, 7, 8, 11, 12, 14, 17, 18, 19, 20, 21, 22, 23, 24, 27, 28, 29, 30, 31, 32, and 33 (Supplementary Figure S1C), we integrated these sub-clusters as *SPINK1*-high HCC cells and integrated the remaining sub-clusters as *SPINK1*-low HCC cells.

Additionally, using identical procedures, we conducted scRNA-seq data analysis in cohort 4. The cell clustering approach identified six clusters, designated as B cell, endothelial cell, fibroblast, hepatocyte, myeloid cell, and T/NK cell (Figure 2D). The expression levels of marker genes aligned with the cell annotation results (Figure 2E), affirming the accuracy of our cell type identification. Subsequently, we focused on the malignant hepatocyte component among all cell types, revealing variable *SPINK1* expression levels in hepatocytes (Figure 2F). Further re-clustering of hepatocytes yielded 38 sub-clusters (Supplementary Figure S2A,B), with sub-clusters 2, 5, 6, 7, 8, 12, 14, 15, 17, 18, 19, 21, 22, 24, 27, 30, 32, 34, and 35 showing high *SPINK1* expression levels (Supplementary Figure S2C). Consequently, we consolidated these sub-clusters into *SPINK1*-high cells, while the remaining sub-clusters were grouped as *SPINK1*-low cells.

### 3.3. Active Drug Metabolism of SPINK1-High Cells

**Figure 3:**
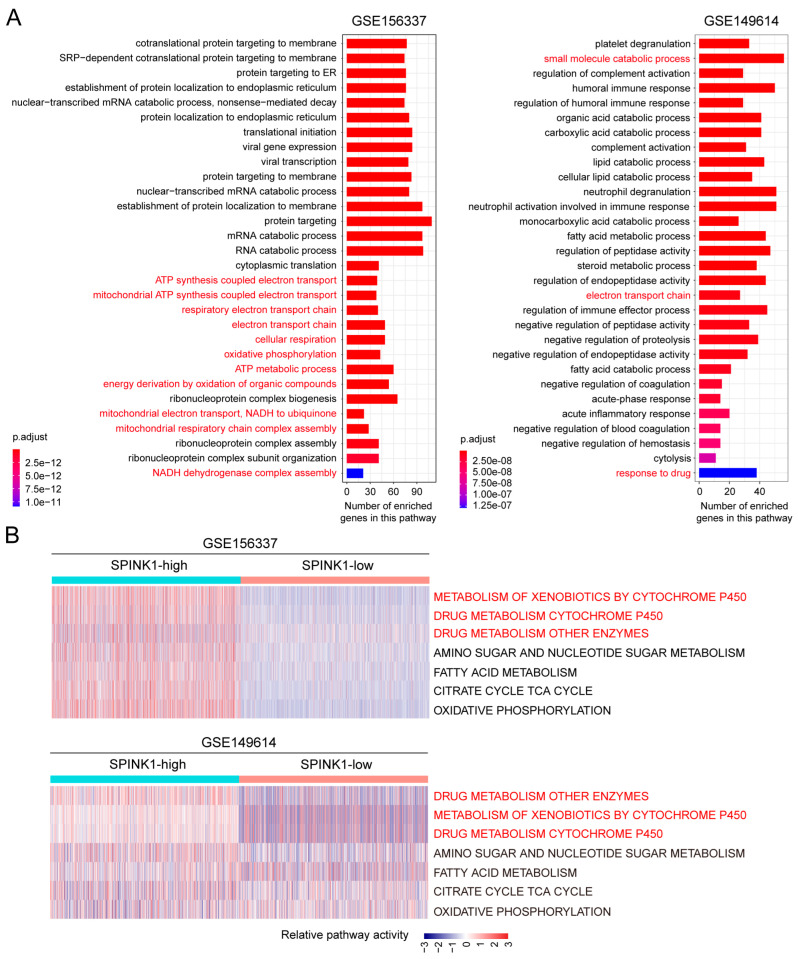
Identification of drug-related metabolic signaling pathways in *SPINK1*-high HCC tumor cells through scRNA-seq data. (**A**) Gene ontology analysis of the DEGs in *SPINK1*-high tumor cells versus *SPINK1*-low tumor cells in cohort 3 (GSE156337) and cohort 4 (GSE149614). Drug metabolism-related signaling pathways are highlighted in red. (**B**) GSVA analysis evaluating the relative activities of representative drug-related metabolic pathways (highlighted in red) in *SPINK1*-high tumor cells versus *SPINK1*-low tumor cells in cohort 3 (GSE156337) and cohort 4 (GSE149614). Both cohorts identified consistent higher activation level of drug metabolic signaling pathways in *SPINK1*-high cells than *SPINK1*-low cells.

To investigate the functions of SPINK1, we examined the differentially expressed genes (DEGs) between *SPINK1*-high and *SPINK1*-low cells in cohort 3 and cohort 4, independently. Signaling pathway enrichment analysis of DEGs in *SPINK1*-high cells from cohort 3 revealed multiple metabolism pathways in the top 30 enriched signaling pathways, including cellular oxidative pathways (Figure 3A, left, highlighted in red). Signaling pathway enrichment analysis of DEGs in *SPINK1*-high cells from cohort 4 identified many drug-related responsive pathways in the top 30 enriched signaling pathways (Figure 3A, right, highlighted in red). These findings suggest that SPINK1 may be involved in drug metabolism in HCC.

To systematically assess the metabolic characteristics of *SPINK1*-high cells, we introduced a 70-pathway metabolic panel [19]. In cohort 3, *SPINK1*-high cells showed higher activation at oxidative phosphorylation pathway, glutathione metabolism pathway, metabolism of xenobiotics by cytochrome P450 pathway, drug metabolism cytochrome P450 pathway, and drug metabolism other enzymes pathway, compared with *SPINK1*-low cells (Figure 3B, upper, and Supplementary Figure S3, highlighted in red). Similarly, *SPINK1*-high cells in cohort 4 also showed stronger activities in drug metabolic pathways, including drug metabolism cytochrome P450 pathway, metabolism of xenobiotics by cytochrome P450 pathway, and drug metabolism for other enzymes, compared with *SPINK1*-low cells (Figure 3B, lower, and Supplementary Figure S4, highlighted in red). Analysis of the metabolic status using another 93-pathway metabolic panel [20] yielded similar results (Supplementary Figures S5 and S6, highlighted in red). Collectively, these findings strongly suggested a drug detoxification function of SPINK1.

**Figure 4:**
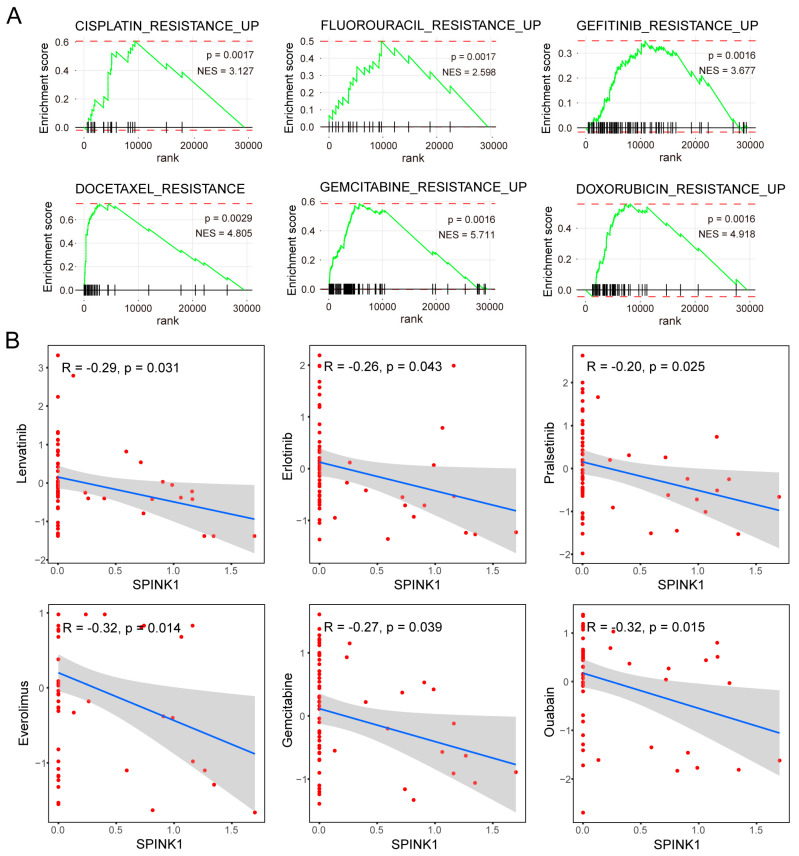
Validation of drug resistance in *SPINK1*-high cells using scRNA-seq data and tumor cell lines. (**A**) GSEA plots illustrating the enrichment of DEGs of *SPINK1*-high cells within therapeutic-resistant pathways in cohort 3 (GSE156337). *p* values and normalized net enrichment score (NES) are indicated on the plots. (**B**) Dot plots demonstrating the negative correlation between *SPINK1* expression levels and drug effectivities, including lenvatinib, erlotinib, pralsetinib, everolimus, gemcitabine, and ouabain in tumor cell lines. The x-axis represents the expression level of *SPINK1* in certain tumor cell line, and the y-axis represents the effectiveness of drugs in this cell line. *p* values and correlation coefficient values (R) are indicated on the plots.

Subsequently, we conducted GSEA on the DEGs of *SPINK1*-high cells and found significant enrichment of these DEGs on multiple drug resistance pathways. In cohort 3, the DEGs of *SPINK1*-high cells versus *SPINK1*-low cells demonstrated enrichment in pathways associated with cisplatin resistance, fluorouracil resistance, gefitinib resistance, docetaxel resistance, gemcitabine resistance, doxorubicin resistance, trabectedin resistance, and tamoxifen resistance (Figure 4A, and Supplementary Figure S7A). In cohort 4, the DEGs of *SPINK1*-high cells versus *SPINK1*-low cells were enriched in cisplatin resistance and tamoxifen resistance pathways (Supplementary Figure S7B). Furthermore, a high level of *SPINK1* expression negatively correlates with the sensitivity of various targeted drugs and chemotherapy drugs, including lenvatinib, erlotinib, pralsetinib, everolimus, gemcitabine, and ouabain (Figure 4B). Collectively, these data indicate resistance to multiple chemotherapy drugs and targeted drugs in *SPINK1*-high HCC cells.

### 3.4. Treatment Resistance of SPINK1-High Cells Revealed by ST

**Figure 5:**
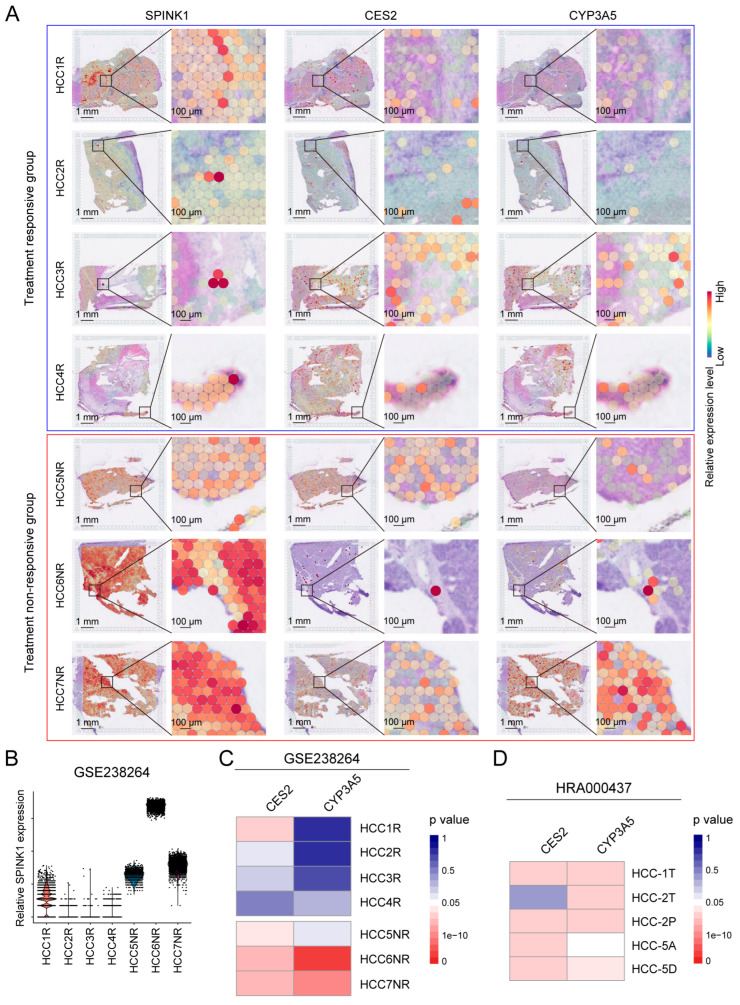
Confirmation and mechanism investigation of treatment resistance in *SPINK1*-high cells through spatial transcriptomics data analysis. (**A**) In situ (left lane) and zoomed-in (right lane) pictures of *SPINK1*, *CES2*, and *CYP3A5* expression in ST chips of treatment-responsive group (blue frame, including HCC1R, HCC2R, HCC3R, and HCC4R) versus treatment non-responsive group (red frame, including HCC5NR, HCC6CR, and HCC7NR) in cohort 5 (GSE238264). *SPINK1* showed co-expression patterns with *CES2* and *CYP3A5* in treatment non-responsive group, rather than treatment-responsive group. R, responsive. NR, non-responsive. (**B**) Relative *SPINK1* expression levels in samples from cohort 5 (GSE238264). The expression level of *SPINK1* was higher in treatment non-responsive group than in treatment-responsive group. (**C**) Statistical analysis of gene co-expression patterns between *SPINK1* and *CES2*, *CYP3A5* in HCC samples from cohort 5 (GSE238264). *p* values of co-expression were colored from red (significant) to blue (non-significant). (**D**) Statistical analysis of gene co-expression patterns between *SPINK1* and *CES2*, *CYP3A5* in HCC samples from cohort 6 (HRA000437). *p* values of co-expression were colored from red (significant) to blue (non-significant).

To probe into the roles of SPINK1 in systematic HCC treatments, we introduced a ST dataset (cohort 5, Supplementary Table S1) collected from HCC patients treated with neoadjuvant cabozantinib and nivolumab [21]. Based on the effectiveness of tumor treatments, patients in cohort 5 were categorized into the responsive group (HCC1R, HCC2R, HCC3R, and HCC4R) and the non-responsive group (HCC5NR, HCC6NR, and HCC7NR). Notably, the expression levels of *SPINK1* were higher in the non-responsive group than in the responsive group (Figure 5A,B), supporting the correlation between high *SPINK1* expression and HCC therapy resistance.

In order to explore the underlying mechanisms of treatment resistance in *SPINK1*-high cells, we first detected oncogenic pathways, and found higher activities of oncogenic signaling, including PI3K/AKT signaling, IL6 signaling, and β-catenin signaling, in *SPINK1*-high cells than *SPINK1*-low cells (Supplementary Figure S8). Given the indicating roles of these signaling pathways on immune checkpoint inhibitor treatment resistance [22,23,24], the activation of these pathways in *SPINK1*-high cells helps to explain the therapy resistance phenotypes. Moreover, we screened all genes in three drug detoxification pathways (drug metabolism cytochrome P450 pathway, metabolism of xenobiotics by cytochrome P450 pathway, and drug metabolism other enzymes) for highly expressed genes in *SPINK1*-high cells compared with *SPINK1*-low cells in cohort 3 and cohort 4. This yielded seven potential regulators, AKR1C1, AKR1C3, CES2, CYP3A5, GSTK1, MGST3, and UGT2B4 (Supplementary Figure S9). Subsequent co-expression analysis between *SPINK1* and these seven regulators was performed (Supplementary Table S2). To facilitate detoxification functions, potential regulators were expected to be correlated with *SPINK1* expression level at the same cell. Therefore, we sought genes that co-expressed with *SPINK1* in the same spot of ST data. The result indicated that *SPINK1* expression levels correlated with the expression of *CES2* and *CYP3A5*, which are critical drug metabolic genes that have been proved to elicit treatment resistance in many types of cancers [25], in most cases in the treatment non-responsive group (HCC5NR, HCC6NR, and HCC7NR) (Figure 5C). This result suggested that *SPINK1*-high cells may facilitate drug detoxification through *CES2* and *CYP3A5*. Additionally, another ST dataset (cohort 6, Supplementary Table S1) [26] was introduced to confirm the co-expression between *SPINK1* and *CES2*, as well as *CYP3A5* (Figure 5D). Collectively, through ST data analysis, *CES2* and *CYP3A5* were identified as potential regulators of therapy resistance in *SPINK1*-high cells.

### 3.5. SPINK1 Overexpression Induces Chemoresistance in HCC

**Figure 6:**
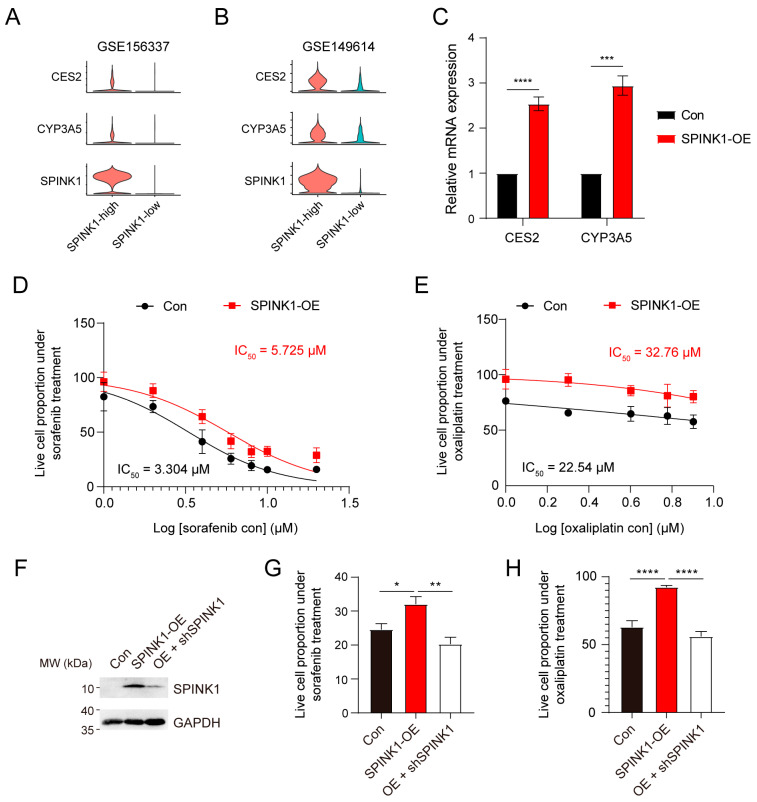
Experimental validations of enhanced drug resistance upon *SPINK1* overexpression. (**A**,**B**) Violin plots depicting the relative higher expression levels of *CES2* and *CYP3A5* in *SPINK1*-high cells compared to *SPINK1*-low cells in cohort 3 (**A**) and cohort 4 (**B**). (**C**) Quantitative real-time PCR illustrating the higher expression levels of *CES2* and *CYP3A5* in *SPINK1*-overexpression cells versus control cells, OE, overexpression, *p* < 0.001 ***, *p* < 0.0001 ****. (**D**,**E**) Sorafenib-resistance curves (**D**) and oxaliplatin-resistance curves (**E**) of control cells (black) and SPINK1-overexpressed cells (red). (**F**) Western blot results displaying the relative protein expression levels of SPINK1 and GAPDH in control cells (Con), *SPINK1*-overexpressed cells (SPINK1-OE), and cells knockdown of *SPINK1* after overexpression (OE + shSPINK1). (**G**,**H**) CCK-8 assay displaying the relative survival proportion of control cells, *SPINK1*-overexpressed cells, and cells knockdown of *SPINK1* after overexpression under 8 μM sorafenib (**G**) or oxaliplatin (**H**) treatment, *p* < 0.05 *, *p* < 0.01 **, *p* < 0.0001 ****.

Considering the elevated expression levels of *CES2* and *CYP3A5* in *SPINK1*-high cells compared to *SPINK1*-low cells (Figure 6A,B), we proceeded to investigate the relationship between *SPINK1* expression and *CES2*, as well as *CYP3A5* expression, using quantitative real-time PCR. Compared with control cells, HCC cell line PLC/PRF/5 overexpressed with *SPINK1* (Supplementary Figure S10) exhibited higher levels of *CES2* and *CYP3A5* (Figure 6C). This suggests that *SPINK1* overexpression induces the expression of *CES2* and *CYP3A5*. Furthermore, to validate the chemotherapy resistance of *SPINK1*-high cells, we used sorafenib and oxaliplatin, two representative first-line drugs for HCC. In comparison to control cells, PLC/PRF/5 cells overexpressed with *SPINK1* exhibited a higher survival proportion under sorafenib (Figure 6D) or oxaliplatin (Figure 6E) treatment, indicating a stronger treatment resistance in *SPINK1*-high cells. Moreover, to further confirm the treatment resistance of *SPINK1*, we used shRNA to knockdown the expression level of *SPINK1* in *SPINK1*-overexpressed cells (Figure 6F) and detected the treatment sensitivities of these cells. The result showed that knockdown of *SPINK1* attenuated the resistance of *SPINK1*-overexpressed cells under sorafenib (Figure 6G) or oxaliplatin (Figure 6H) treatments. These results collectively indicate the chemoresistance roles of SPINK1.

## 4. Discussion

HCC is the third leading cause of mortality among malignancies, presenting a formidable challenge in therapeutic intervention [1]. Current first-line treatment strategies for HCC involve a combination of molecular targeted therapies such as sorafenib and lenvatinib, alongside traditional cytotoxic chemotherapy [27,28,29]. However, the emergence of chemoresistance significantly impedes the efficacy of these treatments. Although immunotherapies, particularly immune checkpoint inhibitors, like anti-programmed death receptor-1 (PD-1) and anti-cytotoxic T-lymphocyte antigen-4 (CTLA-4) antibodies, have shown promise in pre-clinical studies [30], their effectiveness in HCC remains limited, with less than 30% of tumors exhibiting prolonged survival upon PD-1 antibody treatment [31]. The combinational use of PD-1 and CTLA-4 inhibitors in advanced HCC patients has not yet been confirmed in phase III clinical trials [32,33]. Although previous articles have attributed the immune checkpoint blockade resistance to high activities of oncogenic signaling pathways, including PI3K/AKT signaling pathway, IL6 signaling pathway, and β-catenin signaling pathway [22,23,24], our study provides a more accessible effectiveness evaluation method by detecting the expression level of SPINK1 in tumors.

SPINK1 is an oncogenic protein that is overexpressed in various tumors, including HCC [6,7]. Previous investigations into SPINK1 have primarily focused on bulk tumors [16,34]. However, due to the significant inter-tumoral and intra-tumoral heterogeneity of HCC, a detailed exploration of SPINK1 at single cell level is urgently needed. Our study innovatively combines scRNA-seq and ST to investigate SPINK1 functions. The application of scRNA-seq reduces intra-tumoral transcriptomic heterogeneity, thereby classifying cells based on SPINK1 expression levels. High throughput sequencing enables the profiling of metabolic characteristics in *SPINK1*-high cells, leading to the discovery of drug resistance features. To be noted, the scRNA-seq data in our study comprise two HCC cohorts with different sample origins. Specifically, samples in cohort 3 were exclusively derived from primary HCC tumors, whereas samples in cohort 4 encompass primary sites, portal vein tumor thrombus, and metastatic lymph nodes. The considerable diversity in sample origins and characteristics imply substantial transcriptomic differences between these two cohorts. In cohort 3, we observed significantly higher activities of glucose metabolism, lipid metabolism, and amino acid metabolism in *SPINK1*-high cells compared with *SPINK1*-low cells, whereas such differences were less evident in cohort 4. Importantly, regardless of the intra-cohort transcriptomic differences, treatment resistance in *SPINK1*-high cells is consistent in these cohorts, suggesting the reliability of our observation in a wide range of clinical cohorts. Additionally, the use of ST on HCC tissues not only validates scRNA-seq results, but also introduces histological information. Given the dynamic nature of metabolites, sharing chemotherapy drugs and metabolic enzymes within the tumor niche is plausible. Therefore, the 55-μm diameter spots on ST chips serves as ideal units for studying the metabolic microenvironment. In our study, cells in *SPINK1*-high spots share metabolites with surrounding cells, suggesting that seeking drug-resistant regulators in the *SPINK*-high niche using ST data may yield more reliable results than scRNA-seq.

Through a combination of bioinformatics and experimental validations, we have identified SPINK1 as a promising indicator of HCC treatment effectiveness. A high expression level of SPINK1 in HCC cells may predict poor responsiveness to treatments, including cytotoxic chemotherapy drugs, molecular targeted therapeutic agents, and immune checkpoint inhibitors. Conversely, the treatment insensitivity of SPINK1-high cells strongly suggests that targeting SPINK1 could enhance treatment effectiveness.

Comprehensive bioinformatic and experimental analyses have unveiled the co-expression patterns of SPINK1 with CES2 and CYP3A5 in HCC. Mechanistically, SPINK1 overexpression has been found to induce ERK phosphorylation [11]. The activation of ERK signaling pathway subsequently enhances the function of HNF4α [35], a confirmed transcription factor for CES2 [36] and CYP3A5 [37]. In brief, the SPINK1-ERK-HNF4α-CES2/CYP3A5 signaling pathway indicates the co-expression of SPINK1 with CES2 and CYP3A5. Notably, CES2 and CYP3A5 serve as pivotal regulators in drug metabolic pathways across various cancer types [25,38]. Upregulation of CES2 has been found in oxaliplatin resistance cells. Additionally, oxaliplatin resistance could be reversed by CES2 knockdown [39]. These findings suggest that the activation of the SPINK1-CES2 signaling may contribute to oxaliplatin resistance. Furthermore, a transcription variant of CYP3A5 has been found to induce the production of the N-oxide sorafenib and diminishing its treatment efficacy [40], which implies that the activation of the SPINK1-CYP3A5 signaling may lead to sorafenib resistance. Taken together, high level of SPINK1 expression indicates multi-drug resistance mediated through CES2 and CYP3A5.

Collectively, through comprehensive use of scRNA-seq and ST, we have identified the treatment-resistant characteristics in *SPINK1*-high HCC cells, which attributes to the co-expression pattern of *SPINK1* with *CES2* and *CYP3A5*. This study unveils a novel HCC chemosensitivity stratification tool and provides a promising druggable target for HCC treatments.

## 5. Conclusions

In summary, our study integrates scRNA-seq and ST data to elucidate the roles of SPINK1 in HCC treatment resistance. The overexpression of SPINK1 is indicative of a poorer prognosis for HCC patients. At single-cell level, HCC cells exhibiting high *SPINK1* expression demonstrate resistance to therapy through an active drug metabolic signaling pathway, a process facilitated by the histological co-expression of *SPINK1* with *CES2* and *CYP3A5*. The identification of SPINK1 function on HCC therapy resistance provides a potential indicator for treatment effectiveness prediction.

## Figures and Tables

**Table 1:** Analysis of relationship between SPINK1 expression level and clinical pathological features in HCC cohort 2.

Variant	Patient Number	SPINK1- Positive	SPINK1- Negative	χ^2^	*p* Value
Gender				1.179 × 10^−30^	1
Male	43	29	14		
Female	15	10	5		
Age				0.55247	0.4573
≤59	30	22	8		
>59	28	17	11		
Tumor size (cm)				2.436	0.1186
≤5.0	36	21	15		
>5.0	22	18	4		
Differentiation				14.109	0.0008
Well	14	4	10		
Moderate	37	28	9		
Poor	7	7	0		
Microvascular invasion (MVI)				17.584	0.000152
M0	22	8	14		
M1	14	10	4		
M2	22	21	1		
Capsular invasion				1.6982	0.1925
No	30	23	7		
Yes	28	16	12		
Virus infection background				2.8178	0.09322
HBV/HCV	28	16	13		
Cirrhosis	30	23	6		
TNM Stage				14.888	0.001914
1	18	6	12		
2	16	13	3		
3	17	13	4		
4	7	7	0		
TP53 status				0.037406	0.8466
Mutation	27	19	8		
Wild type	31	20	11		

## Data Availability

Bulk proteomics data of HCC are available at https://ualcan.path.uab.edu/analysis-prot.html (accessed on 8 February 2024). The scRNA-seq of HCC are available at the Gene Expression Omnibus (GEO) repository (GSE156337): https://www.ncbi.nlm.nih.gov/geo/query/acc.cgi?acc=GSE156337 (accessed on 8 February 2024), and GEO repository (GSE149614): https://www.ncbi.nlm.nih.gov/geo/query/acc.cgi?acc=GSE149614 (accessed on 8 February 2024). The ST data of HCC are available at GEO repository (GSE107943): https://www.ncbi.nlm.nih.gov/geo/query/acc.cgi (accessed on 8 February 2024), and Genome Sequence Archive for Human repository (HRA000437): https://ngdc.cncb.ac.cn/gsa-human/browse/HRA000437 (accessed on 8 February 2024).

## Supplementary Materials

The following supporting information can be downloaded at: https://www.mdpi.com/article/10.3390/biom14030265/s1, Figure S1: Sub-clusters of tumor cells in cohort 3; Figure S2: Sub-clusters of tumor cells in cohort 4; Figure S3: Full page of the relative metabolic signaling pathway activities of SPINK1-high versus SPINK1-low cells in cohort 3 (GSE156337). Signaling pathways in red are representative drug-related metabolism pathways; Figure S4: Full page of the relative metabolic signaling pathway activities of SPINK1-high versus SPINK1-low cells in cohort 4 (GSE149614). Signaling pathways in red are representative drug-related metabolism pathways; Figure S5: Heatmap showing the relative metabolic signaling pathway activities of SPINK1-high versus SPINK1-low cells in cohort 3 (GSE156337); Figure S6: Heatmap showing the relative metabolic signaling pathway activities of SPINK1-high versus SPINK1-low cells in cohort 4 (GSE149614); Figure S7: Chemotherapy resistance of SPINK1-high cells; Figure S8: GSEA plots showing the enrichment of DEGs of SPINK1-high cells in PI3K/AKT pathway, IL6 pathway, and β-catenin pathway; Figure S9: Violin plots showing the relative expression of drug detoxification regulators between SPINK1-high cells and SPINK1-low cells in cohort 3 (GSE156337) and cohort 4 (GSE149614); Figure S10: Western blot results showing the expression levels of SPINK1 and GAPDH in control cells versus SPINK1-overexpressed PLC/PRF/5 cells.; Table S1: Brief information on the clinical cohorts in our study; Table S2: Co-expression analysis of SPINK1 with drug detoxification regulators.
