# Supplementary material for: SPINK1 Overexpression Correlates with Hepatocellular Carcinoma Treatment Resistance Revealed by Single Cell RNA-Sequencing and Spatial Transcriptomics
Source: Biomolecules. 2024 Feb 22;14(3):265. doi: 10.3390/biom14030265 (PMC10968071; doi:10.3390/biom14030265)
Supplement: Supplementary file 1 [file biomolecules-14-00265-s001.zip › biomolecules-2847993 - supplementary.pdf]

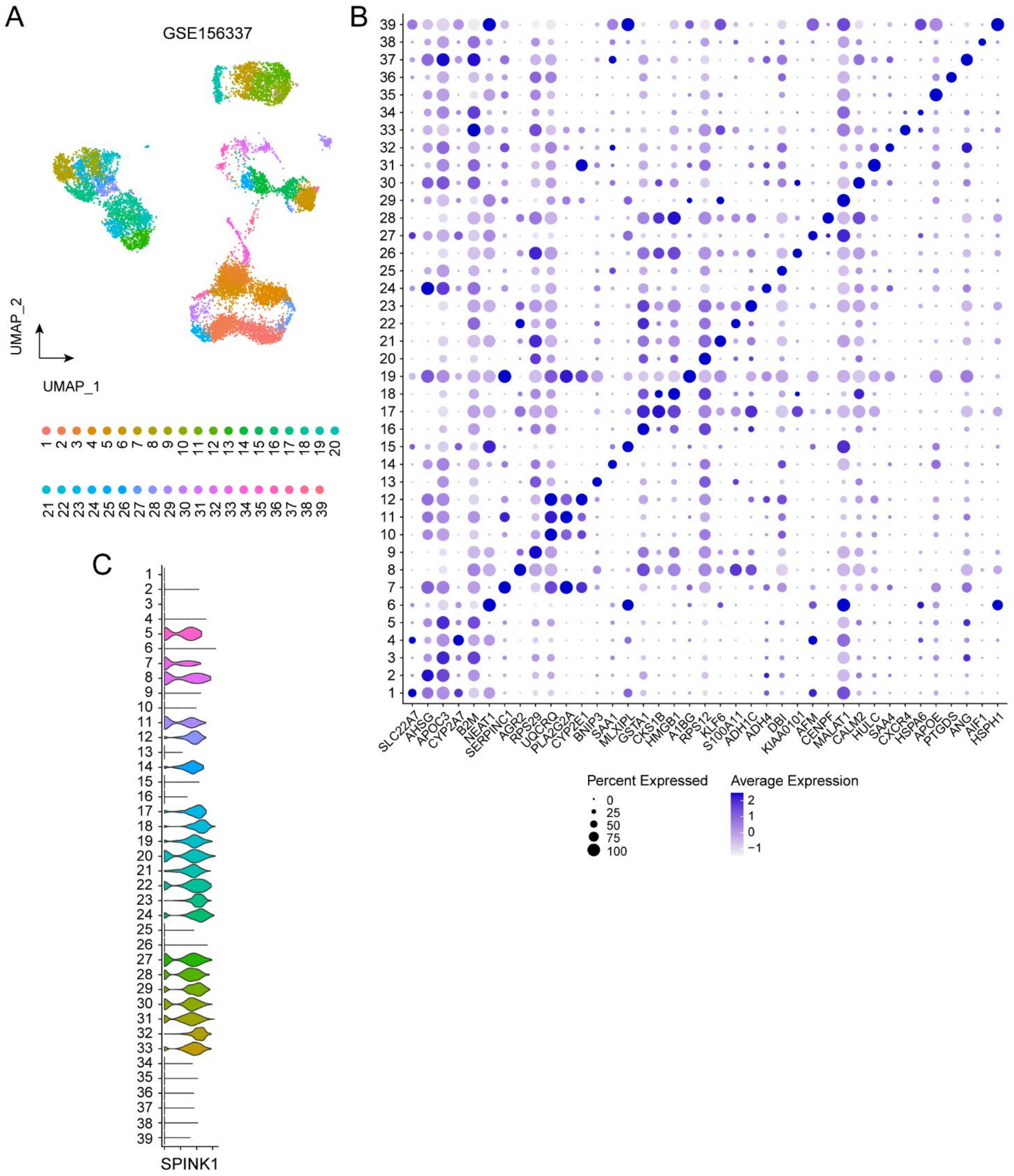

Supplemental Figure S1. Sub-clusters of tumor cells in cohort 3. (**A**) UMAP plot showing sub-clusters of tumor cells in cohort 3 (GSE156337). (**B**) Dot plot showing the marker gene expression levels and proportions in 39 tumor cell sub-clusters of cohort 3 (GSE156337). (**C**) Relative expression levels of *SPINK1* in tumor sub-clusters of cohort 3 (GSE156337).

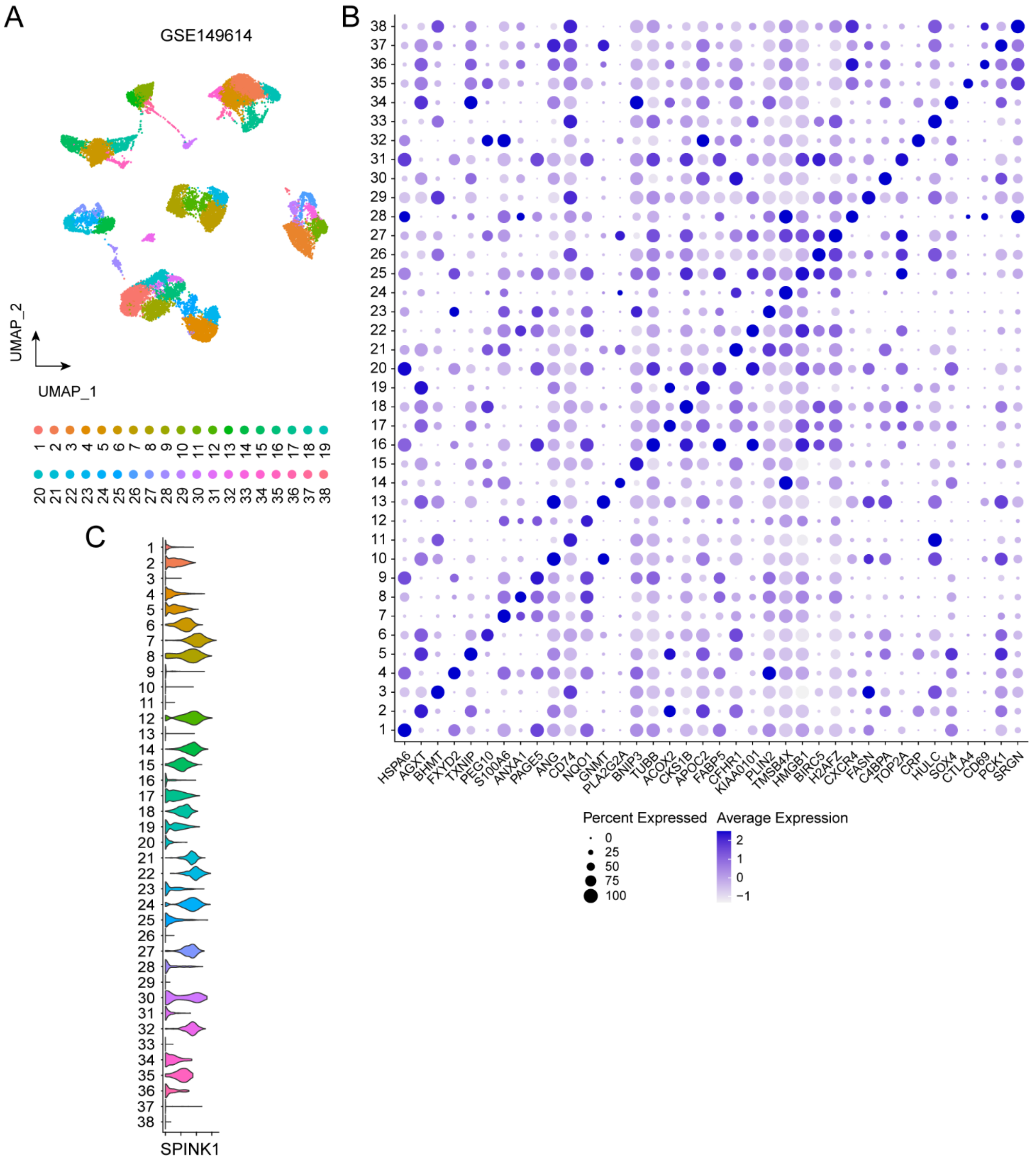

Supplemental Figure S2. Sub-clusters of tumor cells in cohort 4. (**A**) UMAP plot showing sub-clusters of tumor cells in cohort 4 (GSE149614). (**B**) Dot plot showing the marker gene expression levels and proportions in 38 tumor cell sub-clusters of cohort 4 (GSE149614). (**C**) Relative expression levels of *SPINK1* in tumor sub-clusters of cohort 4 (GSE149614).

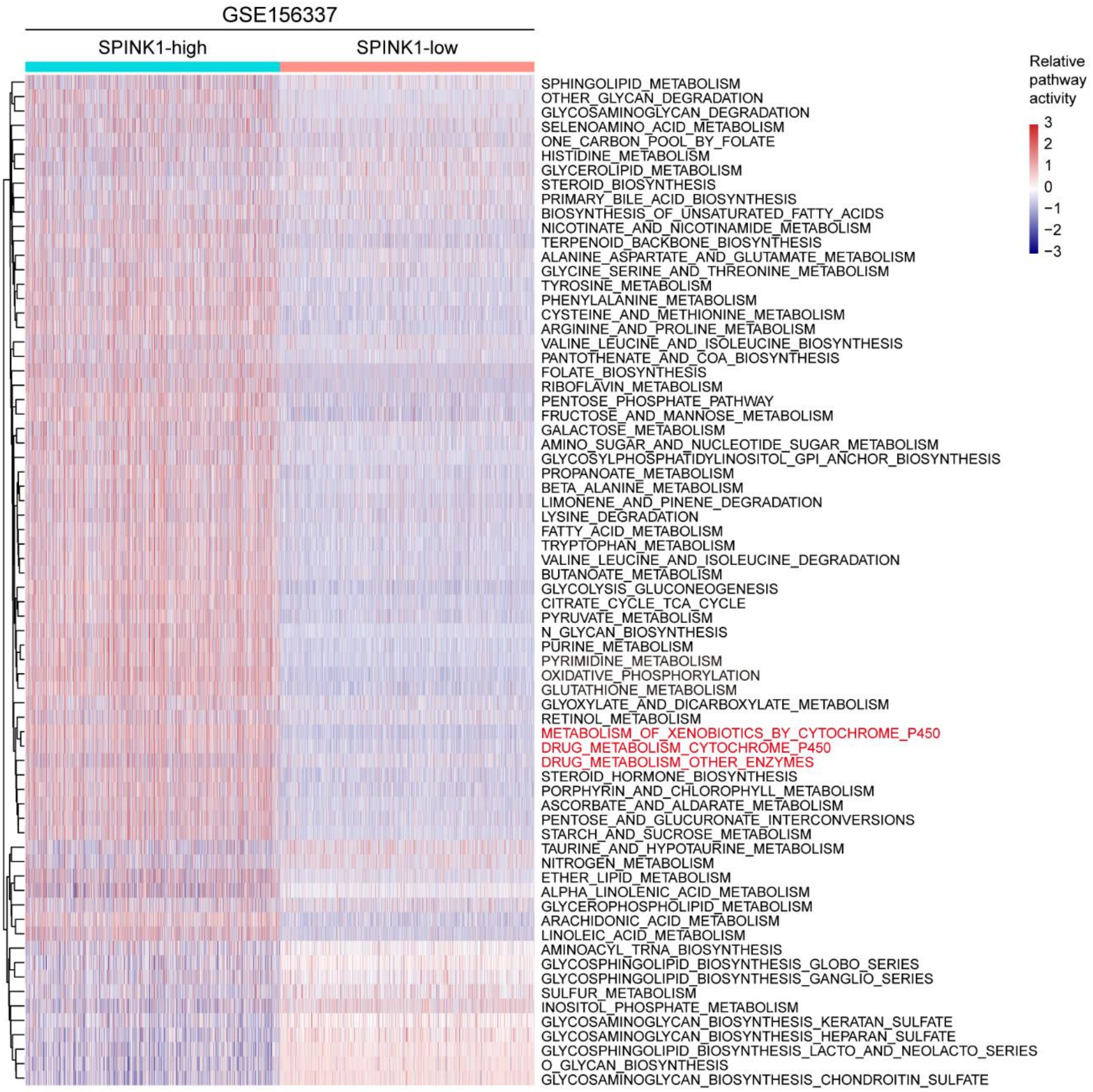

Supplemental Figure S3. Full page of the relative metabolic signaling pathway activities of *SPINK1*-high versus *SPINK1*-low cells in cohort 3 (GSE156337). Signaling pathways in red are representative drug-related metabolism pathways.

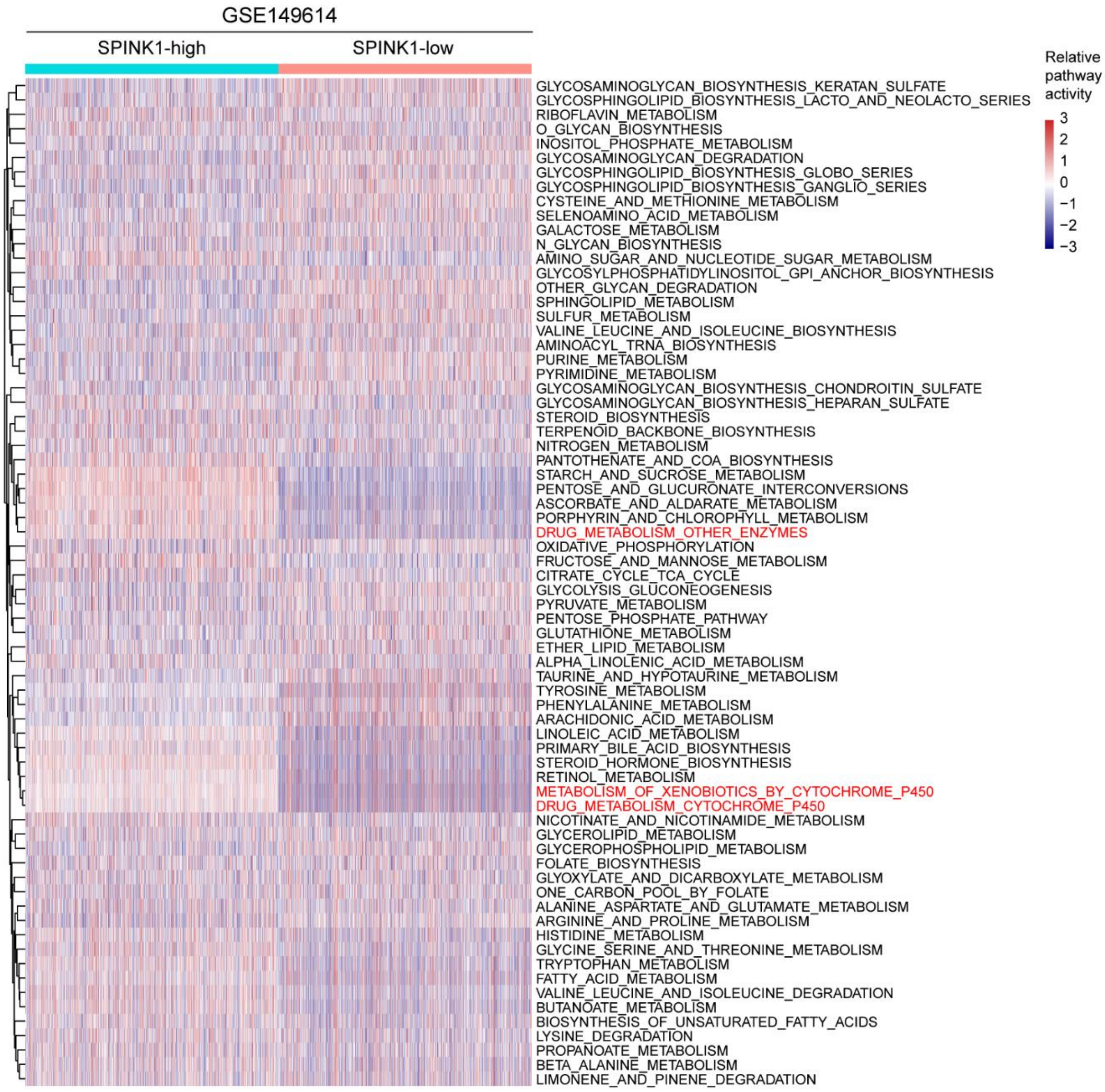

Supplemental Figure S4. Full page of the relative metabolic signaling pathway activities of *SPINK1*-high versus *SPINK1*-low cells in cohort 4 (GSE149614). Signaling pathways in red are representative drug-related metabolism pathways.

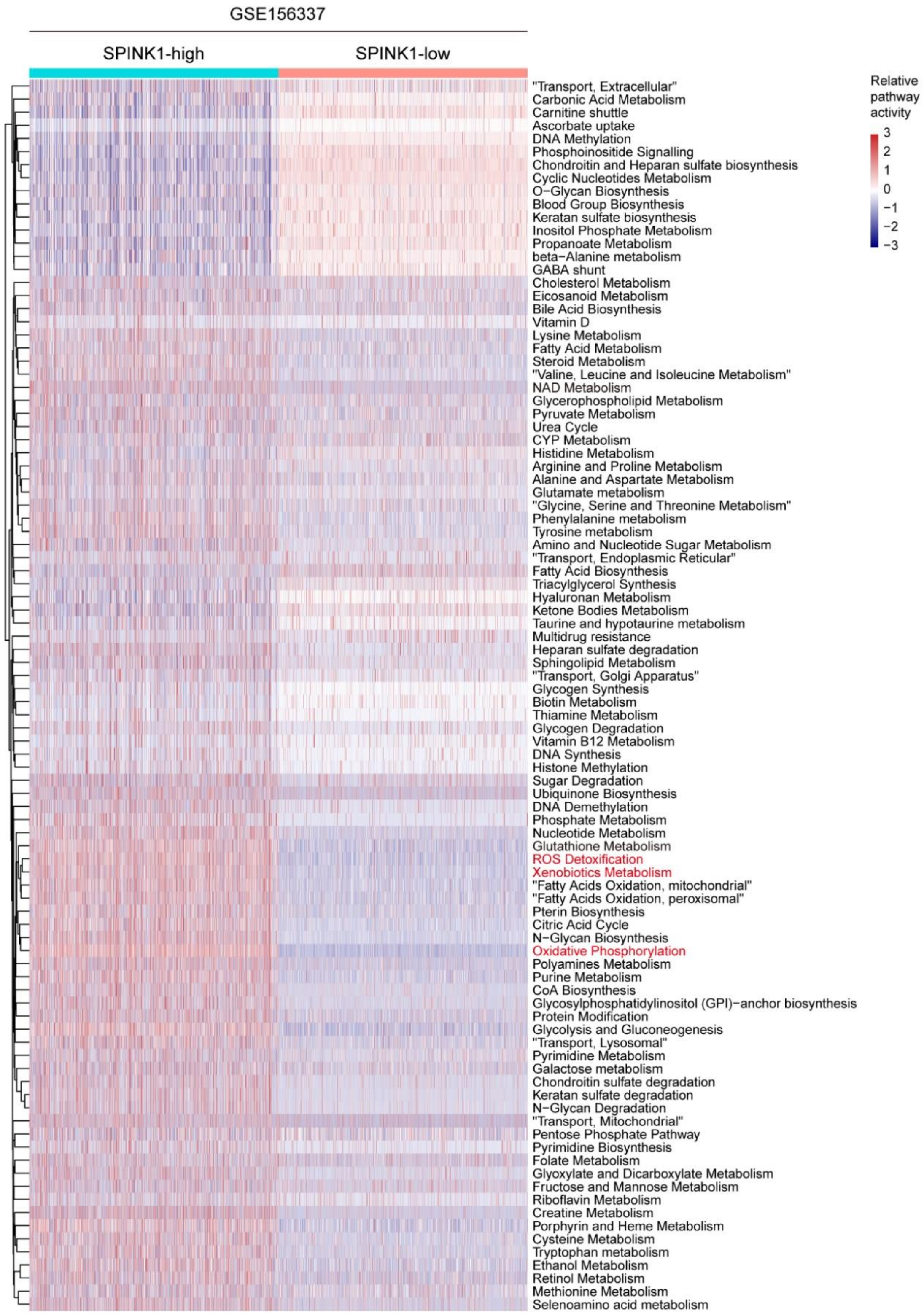

Supplemental Figure S5. Heatmap showing the relative metabolic signaling pathway activities of *SPINK1*-high versus *SPINK1*-low cells in cohort 3 (GSE156337). Signaling pathways in red are representative drug metabolism pathways.

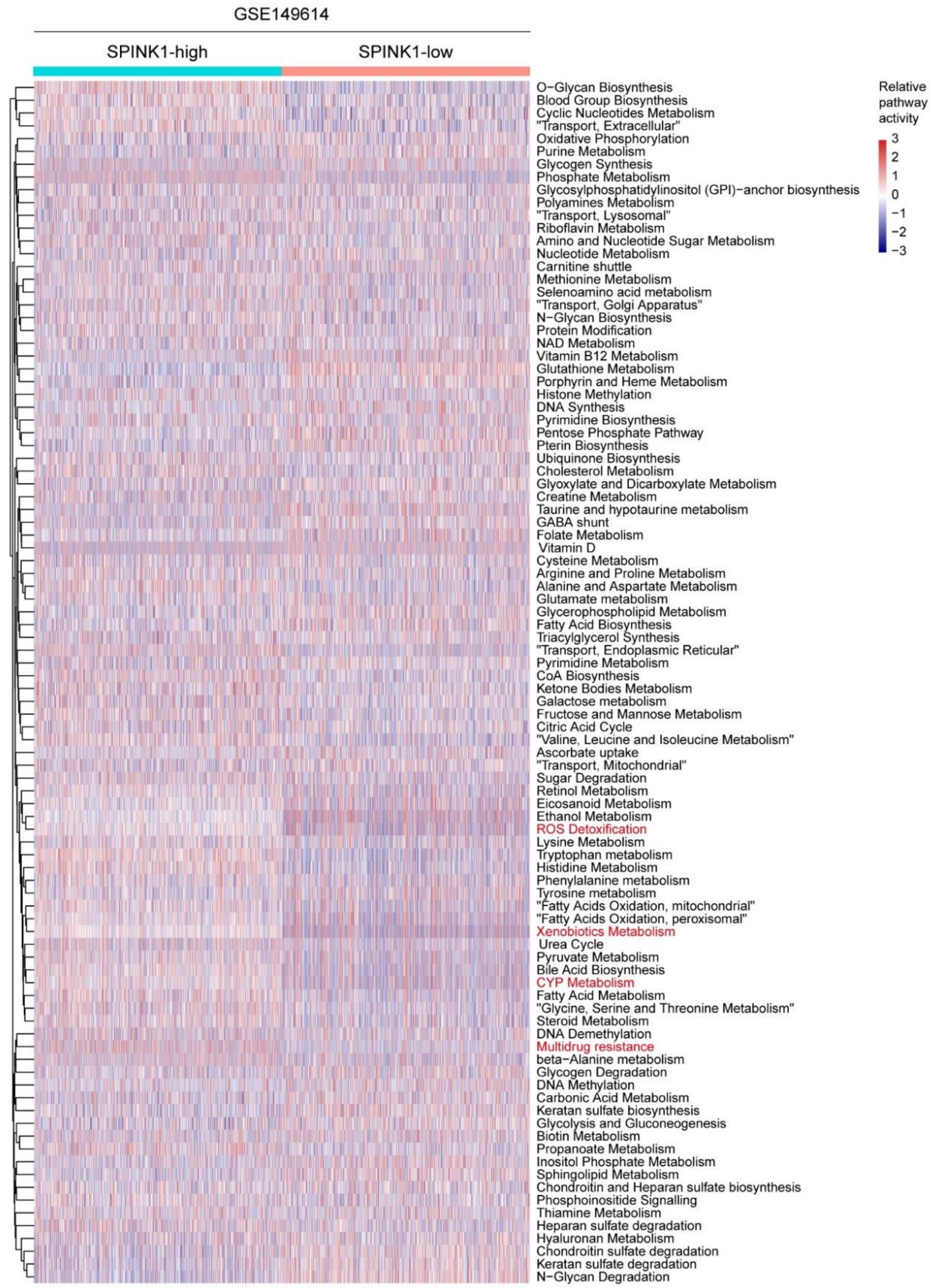

Supplemental Figure S6. Heatmap showing the relative metabolic signaling pathway activities of *SPINK1*-high versus *SPINK1*-low cells in cohort 4 (GSE149614). Signaling pathways in red are representative drug metabolism pathways.

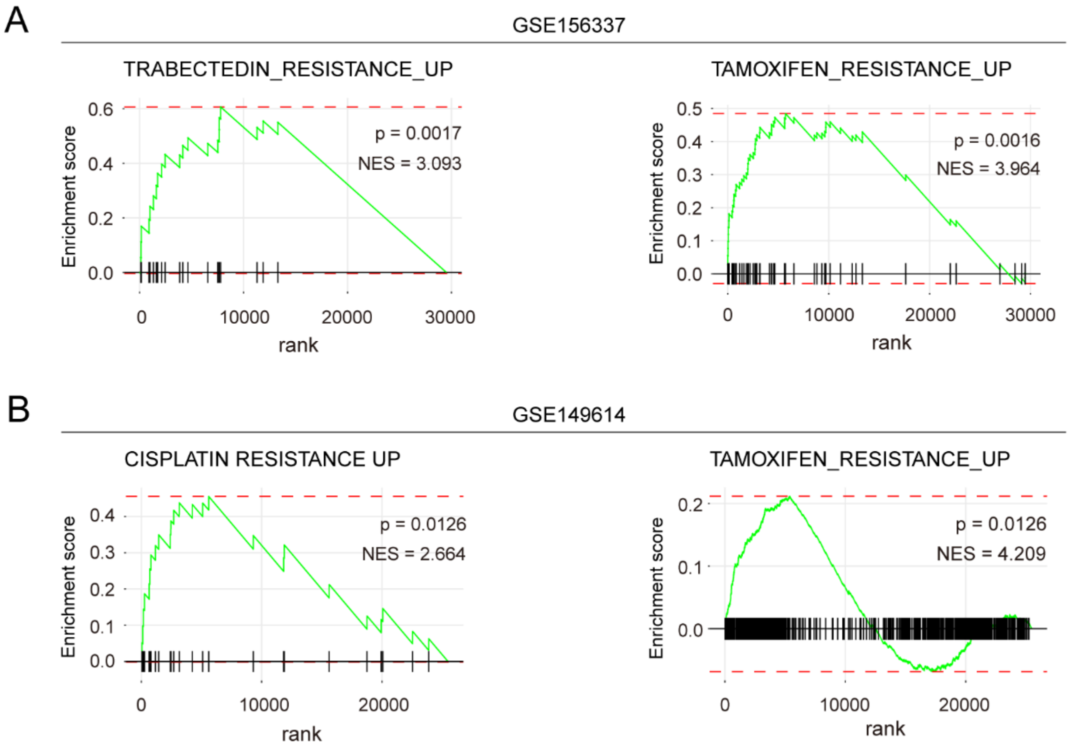

Supplemental Figure S7. Chemotherapy resistance of *SPINK1*-high cells. (**A**) GSEA plots showing the enrichment of DEGs of *SPINK1*-high cells in chemotherapy resistance pathways in cohort 3 (GSE156337). P values and normalized NES are indicated on the plots. (**B**) GSEA plots showing the enrichment of DEGs of *SPINK1*-high cells in chemotherapy resistance pathways in cohort 4 (GSE149614). P values and normalized NES are indicated on the plots.

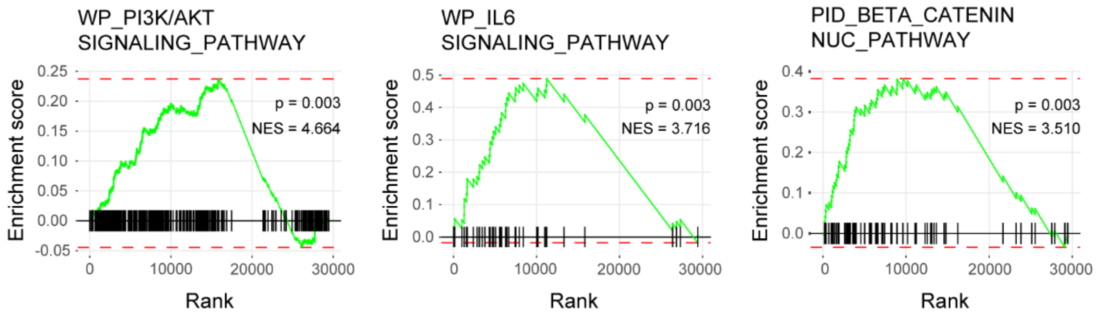

Supplemental Figure S8. GSEA plots showing the enrichment of DEGs of *SPINK1*-high cells in PI3K/AKT pathway (left), IL6 pathway (middle), and β-catenin pathway (right). P values and normalized NES are indicated on the plots.

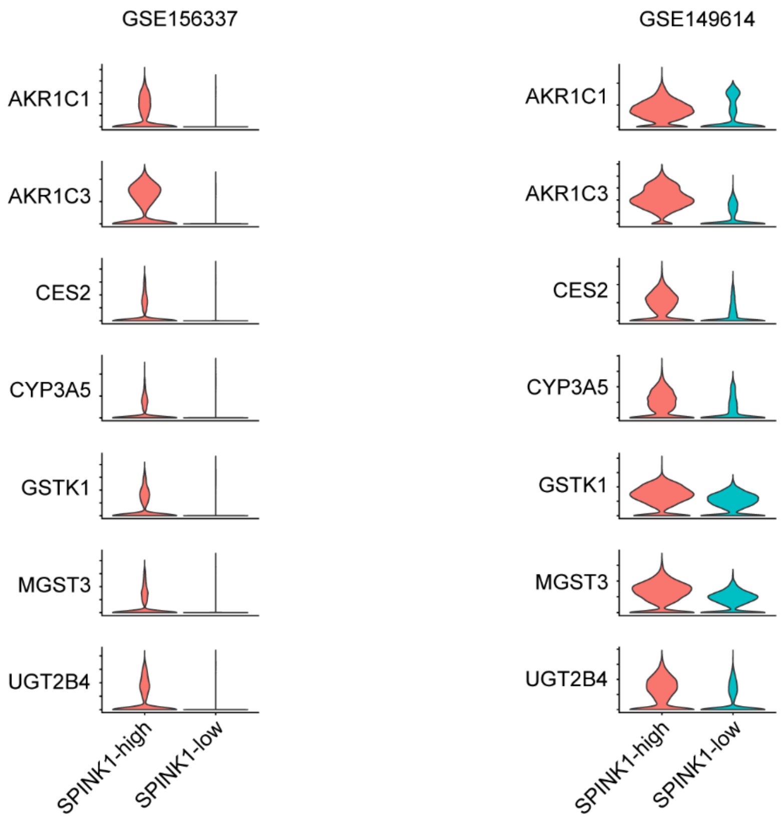

Supplemental Figure S9. Violin plots showing the relative expression of drug detoxification regulators between *SPINK1*-high cells and *SPINK1*-low cells in cohort 3 (GSE156337, left) and cohort 4 (GSE149614, right).

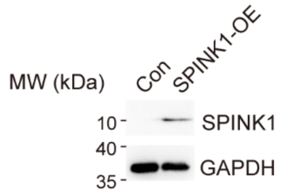

Supplemental Figure S10. Western blot results showing the expression levels of SPINK1 and GAPDH in control cells versus *SPINK1*-overexpressed PLC/PRF/5 cells.

Supplemental Table S1 Brief information of the clinical cohorts in our study.

Number of cohorts	Identifier	Data type	Number of samples	Sample source
Cohort 1	PDC000198, PDC browse	Proteomics data	T (n = 165); N (n = 162)	China
Cohort 2	This study	IHC staining	T (n = 58)	China
Cohort 3	GSE156337, GEO database	scRNA-seq data	T (n = 14)	Singapore
Cohort 4	GSE149614, GEO database	scRNA-seq data	T (n = 10)	China
Cohort 5	GSE238264, GEO database	Spatial transcriptomics	T (n = 7)	America
Cohort 6	HRA000437, GSA human	Spatial transcriptomics	T (n = 5)	China

Supplemental Table S2 Co-expression analysis of *SPINK1* with drug detoxification regulators.

P value	HCC1R	HCC2R	HCC3R	HCC4R	HCC5NR	HCC6NR	HCC7NR
**CES2**	0.004835	0.1117	0.2101	0.5618	0.04854	2.65E-06	3.20E-05
**CYP3A5**	0.8566	0.8818	0.7028	0.3313	0.1212	2.20E-16	1.29E-09
**AKR1C1**	2.20E-16	0.1856	0.8403	0.6348	0.03123	2.20E-16	0.172
**AKR1C3**	8.60E-09	0.2277	0.9968	0.7139	0.1213	2.20E-16	0.01794
**GSTK1**	0.05612	0.998	0.6444	0.3305	0.2242	0.000848	0.2978
**MGST3**	0.3826	0.9602	0.8434	0.3053	0.3458	0.001049	0.8436
**UGT2B4**	2.07E-06	0.7967	0.3054	0.8829	0.9283	2.20E-16	3.76E-08
